# Contact Lens Prescribing Patterns in a University Clinic in Trinidad and Tobago

**DOI:** 10.3390/vision6030055

**Published:** 2022-09-02

**Authors:** Ngozika Esther Ezinne, Kingsley Kene Ekemiri, Gabrielle Nora Harbajan, Anesha Cameisha Crooks, Danquah Douglas, Alex Azuka Ilechie, Khathutshelo Percy Mashige

**Affiliations:** 1 Optometry Unit, Department of Clinical Surgical Sciences, Faculty of Medical Sciences, University of the West Indies, St Augustine Campus, St Augustine 999183, Trinidad and Tobago; 2 Discipline of Optometry, College of Health Sciences, University of KwaZulu-Natal, Durban 4041, South Africa; 3 Department of Optometry and Vision Science, University of Cape Coast, Cape Coast, Ghana

**Keywords:** soft contact lens, rigid gas permeable contact lens, prescribing trend, type of lens, lens design, Trinidad, Tobago

## Abstract

The study assessed the contact lens prescribing patterns and associated factors in a university optometry clinic in Trinidad and Tobago. The data relating to habitual or new contact lens (CL) prescribing patterns among wearers over a two-year period were reviewed. Pearson’s chi-squared test and logistic regression models were used to analyze the findings. The Hosmer–Lemeshow goodness-of-fit test was used to examine the model calibration. A total of 243 CL fits were analyzed, and the Homeshow–Lemeshow goodness-of-fit test indicated a good fit (χ^2^ (7) = 7.296, *p* = 0.399). The mean age of lens wearers was 29.6 ± 12.4 (mean ± SD); the majority, 155 (63.8 %) of whom, were 21 to 40 years old. Most lenses were fitted on females (64.2% of fits overall) and about half of the wearers (*n* = 122, 50.2%) were prescribed lenses for cosmetic purposes. Conventional soft CL were the most prescribed modality of wear, accounting for 129 (53.1%) of the fits. Age from 21 to 40 years was the predictor of lens type prescribed, and those in that age range were four times more likely to be prescribed soft lenses compared to other ages. The patterns of CL prescribing in a university optometry clinic in Trinidad and Tobago are similar to the global market trends with slight variations.

## 1. Introduction

The use of contact lenses (CL) for the management of various ocular conditions, including refractive errors, anisometropia, aphakia, irregular cornea, and keratoconus, has existed for many years [1]. Contact lenses vary in type, design, material, modality of wear, and lens care system [2]. Advances in CL designs and materials recorded globally in the past years could affect CL prescribing patterns, especially with the number of CL wearers increasing to over 140 million [2,3,4]. Over the past two decades, there has been an ongoing evaluation of CL prescribing trends conducted around the world to understand the patterns of CL prescribing better and identify some of the common factors that influence these trends. Studies have shown that females wear CL more than males and soft contact lenses (SCL) for daily wear were the most preferred. Moreover, silicone hydrogel was fitted more often than rigid gas permeable (RGP) CL, especially in developing countries [5]. The socio-demographic characteristics of the population, purpose of wear, prevailing ocular condition, available CL, and the level of education of the optometrists were some of the factors that could influence the prescribing patterns [6].

Worldwide, CL are gaining popularity as an alternative to spectacles for the correction of refractive errors. However, there is still limited information on CL prescribing trends in the Caribbean and in Trinidad and Tobago in particular. Therefore, the purpose of the present study was to assess the current CL prescribing trends in Trinidad and Tobago and assess the factors influencing the prescribing of SCL. The data will aid optometrists and CL practitioners in Trinidad and Tobago in further understanding the local contact lens market and comparing it with other developed markets.

## 2. Method

In this cross-sectional study, the case folders of 243 habitual or new CL wearers at the University of the West Indies (UWI) Optometry Clinic over a two-year from January 2017 to December 2018 were reviewed. The center is well equipped and staffed by highly trained and licensed optometrists. The clinic provides both training and eye care services, including fitting and prescribing CL for patients with keratoconus. Patients visit the clinic from different regions of Trinidad for eye care services. The approval to conduct the study was obtained from the Research and Ethics Committee of the University of the West Indies, St Augustine Campus Trinidad and Tobago, and the study followed the tenets of the Declaration of Helsinki. Furthermore, permission to assess patients’ case files was obtained from the UWI Optometry Unit. The details regarding the demographic profile of each patient fitted in addition to data about the lens type, lens design, purpose of wear, replacement frequency, lens wear regimen, and care systems advised for each patient, over a two-year from January 2017 to December 2018 period were extracted from the patient records and analyzed. Patients who were prescribed CL but had incomplete information in their case files were excluded. The details of the options for each category of lens prescribing are shown in Table 1.

## 3. Data Analysis

All the statistical analyses were performed with the Statistical Package for Social Sciences for Windows (version 21.0; SPSS Inc., Chicago, IL, USA), with confidence intervals set at 95% and statistical significance drawn at an *α* level of 0.05 (two-tailed). Pearson’s chi-squared test assessed the factors associated with the purpose of wear and lens prescribing trend. In the first step of the statistical analysis to assess the factors associated with soft lens prescribing, the influence of age, gender, and purpose of wear with respect to lens prescribing trends (lens type, lens design, and lens material) were analyzed using Pearson’s chi-squared test, and those found to be significant (*p* < 0.05) were included in a multiple logistic regression. Logistic regression assessed the factors influencing prescribing of soft lenses among the study population. Odds ratios (ORs) and 95% confidence intervals were used to assess the strengths of the association, while model calibration was tested with a Hosmer–Lameshow goodness-of-fit test. Small chi-square values (larger *p*-values) (*p* > 0.05) were considered to be well calibrated.

## 4. Results

The data of 243 patients aged 4 to 73 prescribed CL at the UWI optometry clinic from January 2017 to December 2018 were recorded. The mean age of the patients prescribed CL was 29.6 ± 12.4 (mean ± SD); the majority, 155 (63.8%) of the patients, were in the age range of 21 to 40 years. The age distribution was <14 (*n* = 6.25%), 14 to 20 (*n* = 38, 15.6%), 21–40 (*n* = 155, 63.8%, 41 to 50 (*n* = 19, 7.8%), and >50 (*n* = 25, 10.3%). A good number (54.3%) of them reside in rural areas. The majority (42.0%) of the CL wearers were students (Table 1). Most of the lenses were fitted on females (64.2% of fits overall), and most wearers (50.2%) were prescribed lenses for cosmetic reasons. Conventional soft lenses made up 207 (85.2%) of all the CL prescribed. RGP lenses (including corneal, scleral, and orthokeratology lenses) accounted for 36 (14.8%) of the lenses prescribed. Spherical designs were the most prescribed, accounting for 120 (49.4%) of the lenses prescribed. Most of the patients (17.3%) were prescribed contact lenses for “full-time” wear, which is defined as four or more days per week. Silicone hydrogels represent 78 (32.1%) of all soft lenses prescribed. Spherical, toric, and multifocal lenses accounted for 106 (43.6%), 76 (31.3%), and 24 (9.8%) of soft lenses prescribed, respectively. Daily disposables represent the most prescribed lens replacement interval at 147 (60.5%) of all prescriptions, followed by monthly at 38 (15.6%), and yearly at 36 (14.8%). Most of the patients (14%) were prescribed multipurpose care solutions for their reusable CL. The Pearson’s Chi-Square test indicated that there was a significant relationship between the gender of the wearer and the purpose of CL wear (*p* < 0.0001). In univariate (chi-square analysis), a relationship was established between the age group, the purpose of wear (optical and cosmetic), and the material of the lens prescribed (hydrogel or silicone hydrogel) (Table 1).

Gender and the purpose of wear were found to be significantly associated with the type of CL prescribed (soft or hard) (Table 2). No significant association was found between age and the type of CL prescribed. 

Age and the purpose of wear were found to be significantly associated with lens design (spherical or non-spherical) (Table 3). However, no significant association was found between gender and lens design. 

Table 4 presents the binary logistic regression analysis performed to predict the probability that a patient would be prescribed a specific type of lens (soft or RGP) based on their age, gender, and the purpose of wear. The Hosmer–Lemeshow goodness-of-fit test indicated a good fit (χ^2^ (7) = 7.296, *p* = 0.399). The model explained 56.2% (Nagelkerke R^2^ = 0.562) of the variance in the “lens type” prescribed and correctly classified 85.2% of cases. Being aged between “21 and 40” years was the predictor of the lens type prescribed in the study population. The odds of the lens type prescribed for those aged between 21 and 40 years were four times higher than that of the other age groups.

## 5. Discussion

For the first time, we report a CL prescribing pattern from an optometric center in West Indies. The data used for this study were a good representation of the current CL wearing population in the West Indies, as most of the wearers obtain their CL from the study center used for this study.

The results showed that most CL prescribed were SCL. Similar findings were found in other parts of the world [2,7,8]. Globally, soft lenses usually account for more than 90% of lens fits. It is noteworthy that silicone hydrogel lenses accounted for one-third of the soft lenses prescribed. The current rate of silicone hydrogel CL (32.1%) prescribing is an interesting trend that shows an increase in knowledge of the benefits of silicone hydrogel lenses and the latest developments in the market, as well as the availability of newer technologies. Similar findings were recorded by Efron et al. [9] and Ezinne et al. [8] at 60.3% and 96.9%, respectively. Conversely, Moodley [6] and Efron et al. [7] reported silicone hydrogel lenses as the most prescribed over conventional hydrogel CL.

Despite the current market popularity of silicone hydrogel CL, conventional hydrogel lenses still represent a significant proportion of the CL in the global market [9]. The low rate of use of RGP lenses observed in the present study is consistent across studies [6,7,8,9]. The reduction in the use of other CL could be due to a lack of information on the latest development in the CL industry. Furthermore, the lack of experience, knowledge, and skills of the optometrists in the procedure of fitting RGP corneal lenses and scleral lenses for managing highly irregular refractive astigmatism and pathologies such as keratoconus could be the reason for not prescribing them. Daily disposable was the most preferred modality of wear for SCL in our study. Consistent with our findings were reports from Unnikrishnan et al. [1] and Efron et al. [10]. This modality of wear is easy to comply with and requires no lens care system, and that could be the reason for the preference. However, the studies conducted by Haddad et al. [2] and Moodley [6] found that monthly lenses were the most frequently prescribed lenses as compared to daily disposables. A study in Abuja, Nigeria [8] reported that a three-months replacement was the most prescribed modality of lens wear. The variation in the findings could be due to the availability of the lenses by the supplier as well as the affordability of these lenses for patients.

Most of the CL were prescribed to females. This finding is consistent across studies in developing [2,5,11] and developed countries [9,10], with only a few countries reporting this demographic to be outside of the 66% to 72% range. However, studies in Karnataka [1] and Malaysia^11^ recorded a higher percentage of female wearers (79.5 % and 76%, respectively) than what we observed in this study. The higher rate of CL usage by females has been attributed to the higher standard attributed to aesthetics than males [12,13]. 

Majority (68.3%) of CL were prescribed to young adults (range 21–40 years, average age 29.6 ± 12.4). It is reported that the mean age of fitting worldwide was 33.3 ± 14.8 years. The corresponding study in Nigeria and Saudi Arabia recorded similar findings, although this varied greatly from recent studies in Australia and the United Kingdom in which the average at fitting is older than 40 years. The high use of CL in this age group could be due to their occupational needs.

Most CL were prescribed for cosmetic purposes, which is consistent with other studies [6,11,14], including studies in the most successful CL markets. On the contrary, high refractive error was the major reason for prescribing CL in the corresponding study in Nigeria [8] (Table 4).

In terms of the lens design, the data show that spherical designs were the most widely prescribed, and toric lenses accounted for one-third of all the designs prescribed. Similar findings were reported in studies by Moodley [6] and Ezinne et al. [8]. This could be because practitioners may not be skilled or experienced in toric lens fitting and therefore prefer to prescribe spherical lenses for patients with astigmatism. 

Most studies did not specify how long the CL were worn per day and or in a week by their patients, and that made it difficult to compare the current study findings with others. However, the number of hours a CL is worn per day depends on the material, lens type, design, comfort, the modality of wear, and indication for prescribing CL. In the present study, most wearers were prescribed lenses for 7 to 12 h per day in a week, followed by those prescribed for 1 to 6 h per soft day in a week. Consistent with this finding, a study [9] in the United States of America found that nearly all the rigid lens wearers were prescribed lenses primarily for 7 days a week to avoid inconveniences of patients readapting to rigid lenses if not worn full time. Furthermore, in a survey in the United Kingdom [10], it was revealed that 49% of the respondents wore contact lenses every day.

The most prescribed CL care system was the multipurpose solutions. Similar findings were recorded in Jordan and Nigeria [2,8]. This could be because multipurpose solutions are easier to use and have fewer complications when compared to others, such as the hydrogen peroxide system, which is less affordable and has a risk of corneal toxicity. The choice of multipurpose solutions in this study could also be due to the preference of the practitioner and the type of CL prescribed. Moreover, the limited variety of solutions by the suppliers as well, as the fact that various CL companies recommend certain solutions for their lenses, could also influence the choice of the lens care system.

## 6. Limitations

The present study has some limitations. It was susceptible to bias in terms of selection and information because of its retrospective nature. In addition, the small sample size prevented accurate statistical analyses in categories with few subjects. Furthermore, incomplete patient information or missing data associated with retrospective studies reduced the total number of files reviewed, which affected the data analysis. Moreover, the data used in the current study were obtained from one source (UWI Optometry clinic), which might not represent the overall prescribing habits of other optometrists and CL practitioners in Trinidad and Tobago. Despite the limitations, our study was the first to provide an insight into CL prescribing patterns in this region, and the key findings were comparable with results from other studies.

## 7. Conclusions 

The CL prescribing pattern in Trinidad and Tobago follows the global trend. SCL is still the most prescribed in Trinidad, with spherical designs and daily disposable lenses being the most popular. The use of soft toric and RGP lenses is still not popular. Multipurpose solutions are the best care systems of choice in Trinidad. The common factors which were found to affect the prescribing of CL were the patient’s age, gender, and purpose of wear. However, age was the strongest determinant of soft lens prescribing in the present study, which is consistent with findings in previous studies. Further steps should be tailored toward assessing the CL practitioner’s and wearer’s reasons for preferring soft over RGP CL and barriers to not prescribing RGP CL.

## Figures and Tables

**Table 1:** Prescribing rates of contact lens in hydrogel and silicone hydrogel CL materials by age, gender, and purpose of wear.

Variables	Hydrogel CL Frequency (%)	Silicon-Hydrogel CL Frequency (%)	*p*-Value
Gender			
Male (87)	8 (43.7)	49 (56.3%)	0.028
Female (156)	91 (58.3)	65 (41.7%)	
Age group (*n*)			
<14 (6)	1 (16.7)	5 (83.3%)	0.006
14–20 (38)	18 (47.4)	20 (52.7%)	
21–40 (155)	21–40 (155)	60 (38.7)	
41–50 (19)	7 (36.8)	12 (63.2)	
>50 (25)	7 (32)	17 (68)	
Purpose of wear (*n*)			
Refractive correction (92)	52 (56.5)	40 (43.5)	<0.0001
Cosmetic (122)	76 (62.3)	46 (37.7)	
Therapeutic (29)	1 (3.5)	28 (96.5)	

**Table 2:** Prescribing rates of Soft and RGP lenses by age, gender, and purpose of wear.

Variables (n)	Soft Lenses Frequency (%)	RGP Lenses (%) Frequency	*p*-Value
Gender			
Male (87)	68 (78.2%)	19 (21.8%)	0.021
Female (156)	139 (89.1%)	17 (10.9%)	
Age			
<14 (6)	6 (100)	0 (0.0)	0.141
14–20 (38)	30 (79)	8 (21.1)	
21–40 (155)	129 (83.2)	26 (16.8)	
41–50 (19)	19 (100)	0 (0.0)	
>50 (25)	23 (92)	2 (8)	
Purpose of wear (n)			
Refractive correction (92)	85 (92.4)	7 (7.6)	<0.0001
Cosmetic (122)	115 (94.2)	7 (5.7)	
Therapeutic (29)	7 (24)	22 (76)	

**Table 3:** Prescribing rate of spherical and non-spherical contact lenses by age, gender, and purpose of wear.

Variables (n)	Spherical CL Frequency (%)	Non-Spherical CL Frequency (%)	*p*-Value
Gender			
Male (87)	41 (47.1)	46 (52.9)	0.357
Female (156)	62 (39.7)	92 (59)	
Age			
<14 (6)	5 (83.3)	1 (16.7)	
14–20 (38)	17 (44.7)	21 (55.3%)	
21–40 (155)	74 (47.7)	81 (52.3)	
41–50 (19)	3 (15.8)	16 (84.2)	
>50 (25)	6 (24)	19 (76)	
Purpose of wear			
Refractive correction (92)	46 (50)	46 (50)	0.039
Cosmetic (122)	45 (43.1)	77 (63)	
Therapeutic (29)	14 (48.2)	15 (51.7)	

**Table 4:** Logistic regression predicting soft lens prescribing from patient’s age, gender, and purpose of wear.

Variable	Odd Ratio	95% CI	*p*-Value
Age (21–40)	4.35	1.62–11.71	0.004 *
Gender	1.90	0.998–3.60	0.051
Purpose of wear (refractive correction)	0.14	0.043–0.450	0.050

R^2^ = 0.587 (Hosmer–Lemeshow), 0.331 (Cox Snell), 0.446 (Nagelkerke); model χ^2^ 2 = 7.37, *p* = 0.03. CI = confidence interval. * Significant at *p* < 0.05.

## Data Availability

The datasets used and/or analyzed during the current study are available from the corresponding author on reasonable request.
